# Sequencing-based breast cancer diagnostics as an alternative to routine biomarkers

**DOI:** 10.1038/srep38037

**Published:** 2016-11-30

**Authors:** Mattias Rantalainen, Daniel Klevebring, Johan Lindberg, Emma Ivansson, Gustaf Rosin, Lorand Kis, Fuat Celebioglu, Irma Fredriksson, Kamila Czene, Jan Frisell, Johan Hartman, Jonas Bergh, Henrik Grönberg

**Affiliations:** 1 Department of Medical Epidemiology and Biostatistics, Karolinska Institutet, Stockholm, Sweden; 2 Department of Oncology-Pathology, Karolinska Institutet, Stockholm, Sweden; 3 Department of Clinical Pathology and Cytology, Radiumhemmet, Karolinska University Hospital, Stockholm, Sweden; 4 Department of Clinical Science and Education, Karolinska Institutet, Södersjukhuset, Stockholm, Sweden; 5 Department of Molecular Medicine and Surgery, Karolinska Institutet, Stockholm, Sweden; 6 Department of Breast- and Endocrine Surgery, Karolinska University Hospital, Stockholm, Sweden

## Abstract

Sequencing-based breast cancer diagnostics have the potential to replace routine biomarkers and provide molecular characterization that enable personalized precision medicine. Here we investigate the concordance between sequencing-based and routine diagnostic biomarkers and to what extent tumor sequencing contributes clinically actionable information. We applied DNA- and RNA-sequencing to characterize tumors from 307 breast cancer patients with replication in up to 739 patients. We developed models to predict status of routine biomarkers (ER, HER2,Ki-67, histological grade) from sequencing data. Non-routine biomarkers, including mutations in *BRCA1*, *BRCA2* and *ERBB2*(HER2), and additional clinically actionable somatic alterations were also investigated. Concordance with routine diagnostic biomarkers was high for ER status (AUC = 0.95;AUC(replication) = 0.97) and HER2 status (AUC = 0.97;AUC(replication) = 0.92). The transcriptomic grade model enabled classification of histological grade 1 and histological grade 3 tumors with high accuracy (AUC = 0.98;AUC(replication) = 0.94). Clinically actionable mutations in *BRCA1*, *BRCA2* and *ERBB2*(HER2) were detected in 5.5% of patients, while 53% had genomic alterations matching ongoing or concluded breast cancer studies. Sequencing-based molecular profiling can be applied as an alternative to histopathology to determine ER and HER2 status, in addition to providing improved tumor grading and clinically actionable mutations and molecular subtypes. Our results suggest that sequencing-based breast cancer diagnostics in a near future can replace routine biomarkers.

Advances in primary management of breast cancer have resulted in marked survival improvements, mainly due to abide use of different adjuvant therapies together with early detection using mammography^1^,^2^,^3^,^4^,^5^,^6^,^7^. Routine diagnostics, including assays of routine biomarkers (e.g. ER, PR, Ki-67 and HER2) and morphological features (histological grade), are important for selection of adjuvant treatment. However, current routine techniques for measuring biomarkers and morphological features are lacking precision^8^,^9^,^10^,^11^, thus leading to both under- and overtreatment of women with early breast cancer.

Breast cancer is a heterogeneous disease and stratification of patients based on the multivariate molecular phenotype of their primary tumor provides means for prognostication and prediction of the probability to reduce risk of relapse as an effect of adjuvant treatments. The subtypes originally defined by Sørlie *et al*. in 2001 and later further refined in additional studies^12^,^13^,^14^,^15^,^16^,^17^,^18^ are commonly used for stratification of tumors: Basal-like, Luminal A, Luminal B, HER2 and Normal-like. However, molecular subtyping using gene expression signatures has not yet reached wide implementation in the clinic. In current clinical practice this molecular phenotype is based on four biomarkers that are routinely analyzed by immunohistochemistry (IHC): Estrogen receptor alpha (ER), progesterone receptor (PR), Human epidermal growth factor 2 (HER2), and Ki-67. In addition, histopathological characterization is routinely carried out to determine tumor grade (Nottingham Histologic Grade). Additional histopathological information of importance in the planning of adjuvant treatment include tumor size.nodal status and lymphovascular invasion. The molecular subtypes have demonstrated prognostic value^12^,^16^,^17^,^18^ and are associated with therapeutic targets (e.g. ER and HER2) and predictive of reduced risk of relapse after treatment. The PAM50 panel^18^ classifies tumor samples into the intrinsic subtypes with a demonstrated prognostic performance beyond conventional clinical factors^17^,^19^.

Cancer diagnostics based on molecular profiling, particularly DNA- and RNA-sequencing, could improve the precision of cancer diagnosis by providing comprehensive tumor characterization^20^,^21^, likely enabling the opportunity for better management and personalized treatment based on tumor characteristics.

In this study we evaluate to what extent DNA- and RNA-sequencing-based molecular profiling of primary breast cancer tumors can directly replace and augment current routine diagnostic biomarkers. A prerequisite for clinical implementation is cost-efficiency. Therefore we designed a 1.6 Mb pan-cancer panel to enable detection of point mutations, germ-line risk variants and pharmacogenomics SNPs. Additionally, low-pass whole-genome sequencing was performed for the identification of copy number variants. A full profile, also including RNA-sequencing, required in total only data corresponding to 1/5 lane on the Illumina Hiseq 2500, in high-output mode.

Using our full profile, we evaluate to what extent RNA-sequencing (RNAseq) data allow us to predict the status of routine breast cancer biomarkers (ER, PR, HER2 and Ki-67). We also outline how RNAseq data can be used to define transcriptomic grade, as an alternative to the histological grade. Furthermore, we detect clinically actionable somatic alterations, including those in *ERBB2* (HER2) and *BRCA1/BRCA2*, which represent information with the potential to provide direct added value from sequencing-based diagnostics.

## Materials and Methods

### ClinSeq study

The study is based on samples from the Libro1 and KARMA tissue studies. Briefly, Libro1 is a retrospectively enrolled group of patients who underwent surgery between 2001 and 2008 at the Karolinska University Hospital and KARMA is a prospectively enrolled group of patients from the South General Hospital in Stockholm during 2012. For both studies, germline DNA from blood and snap-frozen tumor tissue was available. In total the ClinSeq data set contained 307 individuals with RNAseq data (see Supplemental Fig. 1 for consort diagram), low-pass whole-genome DNA sequencing (0.5× coverage) to determine genomic copy number variants (CNVs) across the genome, and panel DNA-sequencing (150× coverage) of 484 genes in an custom in-house designed pan-cancer gene panel for detection of point mutations, germ-line risk variants and pharmacogenomics SNPs. The panel was designed in June 2013 through extensive literature search. Additionally, the size of the panel was limited to enable 24 samples to be run simultaneously on one Illumina Hiseq 2500 lane in rapid mode. Information on ER, PR, HER2 and Ki-67 as well as histological grade was collected from medical records. The distribution of clinical biomarkers and phenotypes are provided in Supplemental Table 1. Written informed consent was obtained from all subjects. All experimental protocols were approved by the Regional Ethical Review Board in Stockholm (Reference number: 2013/1833–31/2). All experimental methods were conducted according to approved guidelines.

### TCGA breast cancer study

Clinical data and unaligned RNAseq data from the TCGA dataset were downloaded from the TCGA data portal (N = 1073) with approval from the TCGA data access committee (dbGAP project ID 5621). 35 observations were excluded as potential outliers. ER status was available for 739 individuals, PR status for 738 individuals and HER2 status for 731 individuals, which were included in the replication of receptor status prediction. 507 individuals had histological grade (Elston-Ellis) available and were included in the replication of the transcriptomic grade model.

### Tissue and library preparation, sequencing and preprocessing

Briefly, DNA libraries were constructed using ThruPlex-FD (Rubicon Genomics), one aliquot was used for low-pass WGS and one aliquot was used for capture using the EZ SeqCap kit (Roche Nimblegen) as previously described^22^. The capture kit contained 484 genes known to be somatically mutated or associated with germline risk (Supplemental Table 2). Additionally, 82 pharmacogenic SNPs were also included^23^. Sequencing was performed on Illumina HiSeq 2500. WGS libraries were sequenced to on average 0.5x coverage, captured libraries to around 150x average coverage and RNAseq libraries to a median of 33 million read-pairs per library (paired-end 2 × 101 bases). Preprocessing was performed using AutoSeq (https://github.com/dakl/autoseq), which includes best practices pipelines for the respective data types.

### Prediction modeling

Logistic regression models were fitted with ER, PR, HER2 status as response variable and the expression of each corresponding gene as predictor. Ki-67 was modeled by a linear penalized regression model, *elastic-net*^24^,^25^. Molecular subtypes were assigned using the Nearest Shrunken Centroid classifier^26^ using the PAM50 gene set^18^ with parameters estimated from the TCGA dataset, excluding Normal-like subtype as the clinical relevance of this subtype has been questioned^27^. Prediction modeling of histological grade aimed at classifying tumors into ‘high’ and ‘low’ transcriptomic grade (TG), corresponding to histological grade 1 and 3, was carried out using *elastic-net* models^25^, Individual elastic net models were fitted for each subcomponent of the histological grade: mitotic count, nuclear atypia and tubular formation. Each of these models was trained on tumors with a clinical score of 1 or 3 for each respective component. For prediction of transcriptomic grade, the predicted score (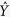) for each component were combined into an overall score defined by the sum over the predictions from each subcomponent model, 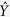
_mitotic (mitotic count), 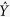
_nuclearity (nuclear atypia), 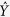
_tubularity (tublar formation). The transcriptomic grade model included 218 genes in total (Supplemental Table 2), 18 of these genes were common with the gene set of 97 genes previously proposed by Sotiriou *et al*.^28^ based on microarray data. To estimate prediction performance in the case of penalized regression models, a nested cross-validation procedure was implemented allowing for unbiased estimation of prediction performance while also optimizing model parameters empirically. Optimization of the amount of penalization (*lambda*) in each elastic net model was optimised in the inner cross-validation, using only the training data from the outer cross-validation. The parameter *alpha*, describing the relative weight between L1 and L2 penalisation was set to 0.5. The prediction performance was estimated using the test set in the outer cross-validation round, i.e. using data that were not involved in any part of the model optimization or parameter estimation. Prediction performance in all prediction models was evaluated using nested cross-validation. Optimal decision boundaries for binary classification problems were determined by the point with minimal distance to the top-left corner of the ROC curve. All statistical analyses were carried out in R^29^. See Supplemental Methods for further details.

### Clinical routine biomarkers

Information on ER, PR, HER2 and Ki-67, as well as histological grade, was collected from medical records. ER and PR status were for most individuals assessed by immunohistochemistry (IHC), classifying tumors that showed staining in 10% or more cells as positive. For a subset of the older samples the radioimmunoassay was used to assess ER and PR status, classifying tumors that had >0.05 fmol/ug DNA as positive. A tumor was classified as HER2 positive if fluorescence *in situ* hybridization (FISH) showed amplification or, in the absence of FISH results, if the sample was graded 3+ by HER2 IHC. FISH was routinely carried out for tumors with >2+ HER2 score determined by IHC. Ki-67 was assessed by IHC and medical records report Ki-67 either as “high”/“low” or as a percent value (% positively stained cells). For the tumors with reported percentage, 20% was considered as the threshold for high proliferation. Grade (Elston-Ellis) was recorded as 1, 2 or 3.

### Histopathological re-examination

Re-examination of ER and HER2 was performed for individuals where the receptor status was discordant between sequencing-based assessment and routine pathology when biobanked material was accessible for re-examination. FFPE archived material was sectioned in 4 um, mounted and stained according to routine protocol at the Laboratory of Clinical Pathology and Cytology at Karolinska University Hospital^30^.

### Actionable mutations

We identified somatic actionable alteration by matching somatic SNVs, indels (insertions and deletions), amplifications and deep deletions to the knowledge database generated by Dienstmann *et al*.^31^. When enumerating patients potentially eligible for targeted drugs, patients were only once counted as a match, and this to the candidate drug with the highest priority (Approved > Late phase studies > Early phase studies).

Please refer to the Supplemental Methods for further details.

## Results

### RNAseq-based prediction of routine biomarker status

**Figure 1:**
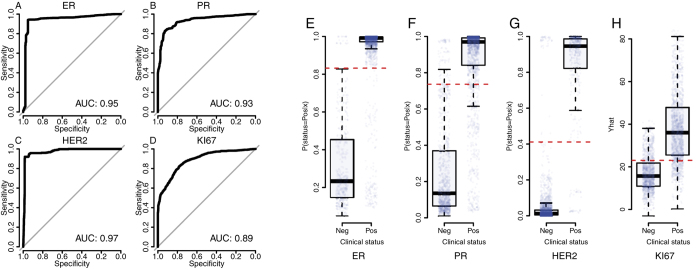
Classification performance of RNAseq-based prediction models for ER, PR, HER2 and Ki-67 as indicated by Receiver operating characteristic curves based on cross-validation for (**A**) ER, (**B**) PR, (**C**) HER2, (**D**) Ki-67. Boxplots of p(status = positive | RNAseq profile) (the probability of positive status of the marker (ER, PR or HER2) given the RNAseq expression profile data) from cross-validation for (**E**) ER, (**F**) PR, (**G**) HER2 and (**H**) predicted (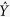) Ki-67 score (% positively stained cells) given RNAseq expression profile data. Dotted red lines indicate optimal decision boundaries as determined by ROC analyses, corresponding to the point on the ROC curve with minimal distance to the top-left corner.

To evaluate if the RNAseq profile can be utilized to predict status of ER, PR, HER2 and Ki-67 we implemented prediction models (see methods) using the clinical status of each of these markers as response variable and RNAseq gene expression variables as predictors. Technical reproducibility was high (Supplemental Fig. 2). Results indicate that prediction of the conventional IHC-based markers^5^ can be achieved with high accuracy; Area under the Receiver Operating Characteristic curve (ROC-AUC) was estimated to 0.95 for ER (95% CI:0.93–0.96), 0.93 for PR (95%CI:0.92–0.94) and 0.97 for HER2 (95%CI:0.97–0.98) (Fig. 1A–C). Status (high/low) of the proliferation marker Ki-67 was predicted with ROC-AUC = 0.89 (95%CI:0.87–0.90) (Fig. 1D). Corresponding decision boundaries for ER, PR, HER2 and Ki-67 are visualized in Fig. 1E,F. AUC estimates for ER, PR and HER2 status were replicated in the TCGA data set; clinical Ki-67 status was not available for analysis. AUC estimates in the TCGA study were similar to those in the ClinSeq study; 0.97 for ER (95%CI:0.95–0.98), 0.92 for PR (95%CI:0.90–0.95) and 0.92 for HER2 (95%CI:0.89–0.96). There were no difference in AUC for ER and PR models between the ClinSeq and TCGA data sets (DeLong’s-test^32^, p-value(ER) = 0.07; p-value(PR) = 0.42). In the case of HER2 the AUC was found to be lower in TCGA study (DeLong’s-test^32^, p-value = 0.004), although the AUC is still to be considered high at 0.92. In this context AUC values >0.9 is to be considered as high and indicative of good prediction performance. In our analyses, AUC values were estimated at ≥0.95 for several of the markers, indicating high prediction performance in general for these markers using RNAseq profiling.

ER and HER2 status have direct implications on the choice of adjuvant treatment. Therefore we have investigated a set of individuals with material available for re-examination and where sequencing-based ER or HER2 status differed from the clinical status (Table 1). ER status was discordant between RNAseq-based calls and clinical ER status in 17 individuals (5.5%). 10 (out of 17) have undergone pathological re-examination where six of ten individuals (60%) were reclassified and concordant with RNAseq-based calls after re-examination (Table 1), indicating that these discordant cases might have been misclassified initially or had an intermediate phenotype. HER2 status was discordant between RNAseq-based calls and clinical HER2 status in eight individuals (2.6%). Out of these, six individuals have undergone pathological re-examination and two out of six individuals (33%) were reclassified compared to initial pathological examination (Table 1). Using the CNV profile (low pass whole-genome sequencing), we investigated the copy number status of *ERBB2* (HER2) for discordant individuals classified as negative by the RNAseq model but positive by IHC/FISH. The CNV data indicate low-grade (ratio close to two) amplifications of the *ERBB2* (HER2) locus on chromosome 17 for these individuals (Supplemental Figs 3–8).

### Transcriptomic tumor grade

**Figure 2:**
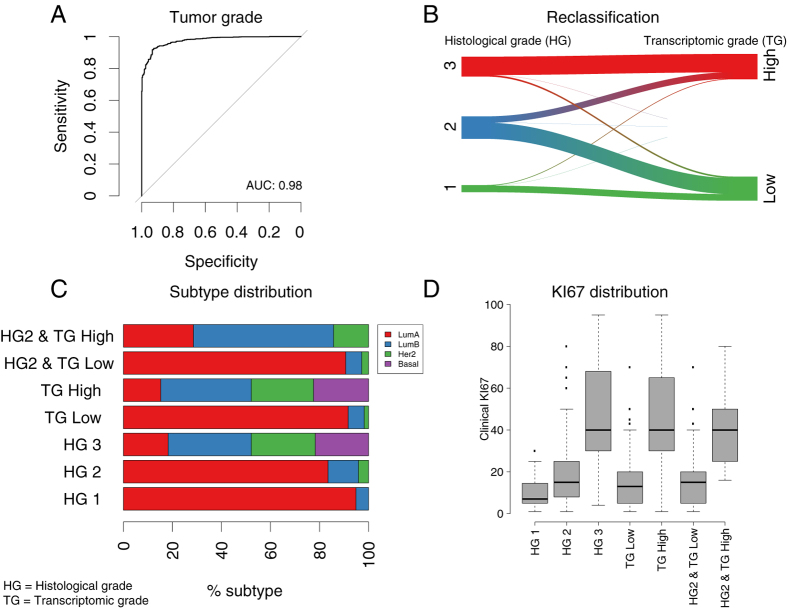
Transcriptomic tumor grade (TG) in comparison with histological grade and molecular subtype. (**A**) ROC curve from cross-validation predictions of histological grade 1 and grade 3 using the TG model. (**B**) Sankey graph of the reclassification of histological grade 1–3 into low TG and high TG. (**C**) Proportion of intrinsic subtypes stratified by histological grade (HG) (1–3) and TG (low, high), where ‘HG2 & TG High’ represents HG 2 tumors reclassified as TG High, and ‘HG2 & TG Low’ represents HG 2 tumors reclassified as TG Low. (**D**) Clinical Ki-67 scores (% positively stained cells) for histological grade (HG) (1–3) and TG (low, high).

With the aim of improving stratification of patients by tumor grade, we applied a RNAseq-based multivariate prediction model to classify tumors into ‘high’ and ‘low’ transcriptomic grade (TG), corresponding to NHG 1 and 3. The model could classify grade 1 and grade 3 tumors with high accuracy (ROC-AUC = 0.98, 95% CI:0.97–0.98, Fig. 2A), indicating that the RNAseq based transcriptomic grade model can be utilized to correctly distinguish between histological grade 1 and grade 3 tumors. TG classification performance was replicated in the TCGA data set, confirming good classification performance (ROC-AUC = 0.94, 95% CI:0.91–0.97), but lower than in the ClinSeq study (DeLong’s-test^32^, p-value = 0.008). Next, we applied the model to reclassify grade 2 tumors in the ClinSeq study (N = 121) into high and low TG (Fig. 2B), since grade 2 status is not informative for clinical decision-making. 14(12%) of grade 2 tumors were reclassified as high TG, and 107(88%) as low TG. Figure 2B shows the reclassification patterns of all tumors in the study using the TG model. We observed no significant difference in subtype distribution (Fig. 2C) between high TG (TG High) and histological grade 3 (HG 3) (Fisher’s exact text, p-value = 0.85), and not between low TG (TG Low) and histological grade 1 (HG 1) (Fisher’s exact text, p-value = 0.86). This indicate that on a molecular subtype level the HG 1 and TG Low groups are similar, and the HG 3 and TG High groups are similar, suggesting the the TG model stratify patients in a way that is consistent with histological grade. We also found that histological grade 2 tumors (HG 2) that were reclassified as low TG (Fig. 2C, HG2 & TG Low) had highly similar subtype distribution as histological grade 1 (HG 1), both dominated by Luminal A subtype, indicating that these reclassified tumors resembles the group of HG 1 tumors, suggesting that these reclassified HG 2 tumors on a molecular subtype level resembles the HG 1 group. Similarly, histological grade 2 tumors (HG 2) reclassified as high TG (Fig. 2C, HG2 & TG High) had a similar subtype distribution as histological grade 3 (HG 3), with a minority of Luminal A and domination of Luminal B and Her2 subtypes (we note that the HG2 & TG High group has no presence of Basal-like subtype tumors since no Basal-like subtype tumors were initially present in the HG 2 group), again confirming that the TG model stratify patients in such a way that the distribution on the molecular subtype level is similar to histological grade. Next, we assessed the distribution of the clinical proliferation marker Ki-67 across histological grades and transcriptomic grades (Fig. 2D). We found that low TG and histological grade 1 displayed similar Ki-67 score distributions, albeit with a small but significant difference in mean scores (two-sided t-test, p-value = 0.004). High TG and histological grade 3 displayed similar Ki-67 scores distribution, with no significant difference in mean scores (two-sided t-test, p-value = 0.81). Altogether, these results suggest that the TG model has the potential to provide means for improved stratification of breast cancer patients by enabling a molecularly consistent reclassification of HG 2 patients into TG low and TG high.

### Somatic mutations in ERBB2 (HER2)

**Figure 3:**
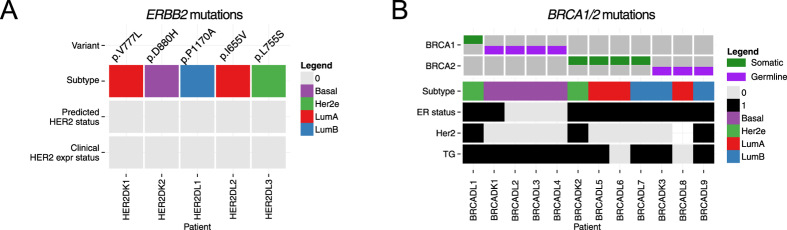
(**A**) Overview of HER2 status, molecular subtype and specific ERBB2 mutation for those individuals with detected *ERBB2* (HER2) somatic mutations. All tumors with somatic mutations in *ERBB2* (HER2) were found to be HER2-negative according to both routine pathology and RNAseq-based classification, indicating normal HER2 expression levels. (**B**) Overview of transcriptomic grade, ER status, HER2 status, molecular subtype and BRCA1/2 mutations status of those individuals with detected germline or somatic mutations in either *BRCA1* or *BRCA2*. Tumors in patients with germline alterations in *BRCA1* are predominantly of the Basal-like subtype. (Key: TG = transcriptomic grade. Status: 0 = negative/low; 1 = positive/high).

Next, we evaluated to what extent somatic mutations in *ERBB2* were present and detectable. Somatic mutations in *ERBB2*(HER2) have the potential to activate HER2 signaling without increased HER2 protein expression^33^, giving them no benefit from trastuzumab. Instead, small-molecule tyrosine kinase inhibitors such as lapatinib and neratinib have proven successful in model systems^33^ and have great potential to be of benefit for these patients, also indicated by preliminary results in humans^34^. We utilized panel DNA sequencing to ascertain mutations in ERBB2. In our study, five patients harbored somatic alterations in *ERBB2*(HER2), all at different positions (Fig. 3A). All but one (P1170A) of the mutations were included in COSMIC^35^, and two (L755S, V777L) were previously described as activating^33^. All of these patients were HER2 negative by routine IHC, indicating a normal level of HER2 protein expression. One of these individuals (RDL6) was predicted to have a HER2-enriched molecular subtype. The group of patients harboring *ERBB2* mutations would normally not have been identified in the routine clinical setting, while DNA-sequencing in this case enable identification of these patients, which could have benefit from alternative treatments, as outlined above.

### Somatic and germline mutations in BRCA1 and BRCA2

Furthermore, we applied panel DNA sequencing to investigate germline and somatic mutations in *BRCA1* and *BRCA2*, due to their potential to impact both treatment and patient follow-up. In total, 12 patients harbored mutations in these genes (*BRCA1*; one somatic, four germline, *BRCA2*; four somatic, three germline) (Fig. 3B). All patients with germline mutations in *BRCA1* (but none of the *BRCA2*-carriers) were predicted to carry a tumor with a basal-like subtype and the variants were all listed as known pathogenic in ClinVar^36^.

### Other actionable somatic alterations

**Figure 4:**
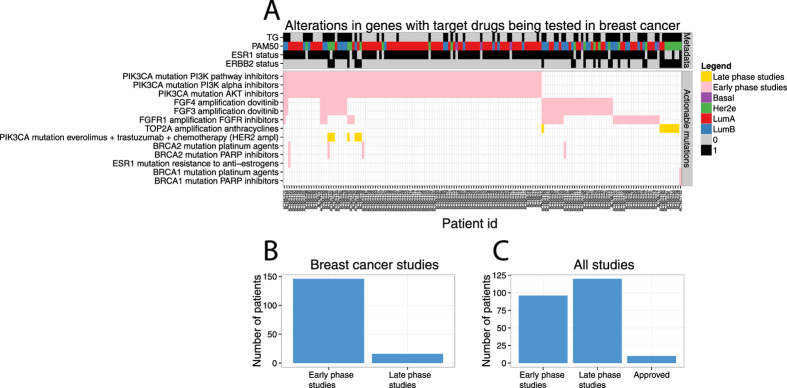
Overview of somatic alterations that are potentially actionable in the ClinSeq study. (**A**) Each patient’s mutational profile was matched to the Dienstmann *et al*. knowledge base of actionable mutations including only breast cancer trials, excluding the ERBB2 amplification. Presence of a colored block in the intersection between an actionable mutation (rows in the heatmap) indicate a match between the somatic alterations in A patient (columns in the heatmap), Studies are classified as “Early” or “Late” from the Dienstmann *et al*. knowledge base^31^, also indicated by color. The top panel of display transcriptomic grade status (“TG”), molecular subtype (“PAM50”), ER status (“ESR1”) and HER2 status (“ERBB2”). (**B**) Summary of the number of patients in the present study that were potentially eligible for targeted drugs for indication breast cancer stratified by early and late phase studies. (**C**) Summary of the number of patients in the present study that were potentially eligible for targeted drugs for any indication, stratified by early and late phase studies as well as approved treatments.

Through the application of panel DNA sequencing and low-pass whole genome DNA sequencing (CNV profiling) 162 patients (53%) in the ClinSeq study (Fig. 4A) were found to have potentially actionable genetic alterations that matched with 13 breast cancer studies on experimental agents under clinical investigation according to the knowledge base by Dienstmann *et al*.^31^. Out of these 162 patients, 107 (35%) harbored SNV or small indel alterations and 72 (23%) CNV alterations, indicating the relevance of profiling both somatic mutations as well as establishing a CNV profile. The most frequent actionable alteration is mutation in *PIK3CA*, for which three types of therapies are under clinical investigation in “early phase studies” (PI3K pathway inhibitors, PI3K alpha inhibitors and AKT inhibitors), and one in a “late phase study” (combination of everolimus, trastuzumab, chemotherapy for HER2-positive patients). Further, *FGF3* and *FGF4* amplification was detected in 42 patients with a perfect overlap. These genes both reside within 10 000 bases, next to each other on chromosome 11, explaining the co-occurrence. These patients could potentially benefit from dovitinib^37^, although current evidence of efficacy is restricted to hormone-receptor positive disease. One “late phase” breast cancer study, according to Dienstmann *et al*., showed an increased pathological complete response in the neoadjuvant setting when treating HER2-positive patients also harboring an amplification of *TOP2A* with anthracyclines^38^. In our ClinSeq study, nine HER2-positive patients harbored amplification in *TOP2A*. Another “late phase” breast cancer study^39^, showed HER2-positive patients with a hyperactive PI3K pathway, defined by somatic mutations in *PIK3CA*, to benefit from the addition of everolimus to their therapy regimen. In the present study, seven HER2-positive patients had somatic mutations in *PIK3CA*. The great majority of patients with actionable mutations in our Clinseq study had alterations in genes matching breast cancer studies in early phase, 146 (48%), while 16 (5%) were matched with studies in late phase (Fig. 4B). If all cancers in the Dienstmann knowledge base were considered, 96 (31%) patients had alterations in genes matching early phase studies, 120 (39%) late phase studie, and 10 (3%) were approved therapies for other cancers (Fig. 4C, Supplemental Fig. 9). DNA sequencing enabled us to detect 162 patients (53%) harboring potentially actionable genetic alterations, indicating a broad potential for providing added value through sequencing-based diagnostics in the future.

## Discussion

Sequencing-based diagnostics is currently being broadly introduced in the clinical setting as a tool to screen for potentially actionable mutations in patients with metastatic disease. It is expected that sequencing-based diagnostics also will be implemented in the diagnostic, non-metastatic setting, assuming it performs at least as good as current routine diagnostics and provides some added value, while also remaining cost efficient. Provided the continuous reduction in sequencing costs over time, sequencing-based diagnostics is expected to be cost efficient in the near future, and may even provide a more cost effective alternative than current routine diagnostics eventually. The goal with this study was to explore if a DNA- and RNA-based sequencing profile could fulfill these criteria, with a focus on ascertaining to what extent sequencing-based diagnostics can be applied as an alternative to current routine diagnostics and to what extent added value is generated.

First, we demonstrated that RNAseq-based models could predict ER, PR and HER2 status in high concordance with routine clinical markers, indicating that RNAseq-based molecular characterization of these biomarkers has the potential to be translated to the clinic in the future. The great benefit of sequence-based breast cancer diagnostics is, however, in providing an improved transcriptomic grade model that stratify patients into two distinct groups, and in providing information on additional targetable somatic alterations and information on somatic alterations affecting drug metabolism, which may be of importance for dosing the patient correctly or avoiding certain compounds. Together these are substantial advantages compared with standard histological management. The main benefits and potential of sequencing-based diagnostics are dependent upon implementing and utilizing a wider range of molecular phenotypes (e.g. subtypes, transcriptomic grade, somatic mutations) in the clinical setting, while we see little benefit in implementing sequencing-based molecular profiling to merely determine the status of current generation routine markers (e.g. ER, PR, HER2), which are currently assayed cost effectively using e.g. immunohistochemistry.

The sequencing-based diagnostic approach also provides multiple other advantages in that it is quantitative, highly reproducible, objective and amenable to automation. In contrast, routine histopathology is semi-quantitative and dependent on subjective human interpretation. Classification of ER, PR and HER2 status based on gene expression (qRT-PCR) has previously been assessed with estimated ROC-AUC^17^ in similar range to that observed here, suggesting that irrespective of technology platform (qRT-PCR or RNAseq), classification of receptor status from gene expression profiling provides highly concordant results compared with routine biomarkers.

Histopathological re-examination of a subset of tumors that were discordant in ER or HER2 status between routine biomarkers and sequencing-based diagnostics revealed that a majority (6/10) of discordant ER individuals were reclassified so that ER status became concordant after reclassification. In the case of HER2 discordant individuals, two out of six of discordant and re-examined cases were reclassified during reexamination. The CNV profile for HER2 discordant individuals revealed that those classified as HER2 negative by RNAseq, while positive by IHC/FISH, all had low-grade amplifications of chromosome 17 (Supplemental Figs 3–8), as determined by low-pass whole genome DNA sequencing. The efficacy of trastuzumab in breast cancer with low-grade amplification of *ERBB2* is currently being evaluated^40^. In one of the HER2 discordant individuals, the laterality of the profiled tumor could not be uniquely matched with the clinical information, which might explain discordance in this patient. Furthermore, the re-examination was not fully blinded and the number of re-examined tumors was limited, therefore these results should be interpreted with caution.

Histological grade is routinely used for patient stratification, particularly to inform treatment decisions regarding adjuvant chemotherapy. We utilized RNAseq data and multivariate modeling to stratify patients into low and high transcriptomic grade, and demonstrated a high concordance for classification of histological grade 1 and grade 3 individuals. The similarities in subtype and Ki-67 distributions indicate that the TG model stratified patients into two groups with highly similar characteristics compared to histological grade 1 and 3. Histological grade 2 is considered an intermediary group, which does not provide clinically actionable information^41^. We were able to re-classify histological grade 2 tumors into low and high transcriptomic grade, illustrating how transcriptomic grade can add clinically useful information for the group of patients classified as histological grade 2. A similar approach has also been proposed previously based on gene-expression data from microarrays^28^,^42^.

DNA sequencing was applied to detect somatic mutations and copy number alterations in key genes, including *ERBB2*(HER2) and *BRCA1/2,* and to assess the proportion of patients in the present study that harbored somatic alterations that may be clinically actionable in the future, as defined by ongoing or concluded clinical breast cancer trials. DNA sequencing allowed detection of somatic mutations in *ERBB2*(HER2) that may warrant targeted treatment. *ERBB2* activating mutations may result in constitutive activation rather than increased expression levels. Therefore, the molecular subtype calls may not identify tumors that are driven by *ERBB2*(HER2) signaling. We also investigated *BRCA1* and *BRCA2* mutation status. Knowledge of *BRCA1/2* germline status could impact the choice of primary surgery. If a patient is a carrier then more radical surgery, and perhaps preventive surgery of the unaffected breast, are potential options. Furthermore, patients with mutations in the BRCA genes are candidates to be included in studies evaluating PARP-inhibitors in the adjuvant setting. BRCA-screening provides additional benefits in providing opportunity to refer carriers to genetic counseling.

In this study 25 patients (8.1%) were reclassified in respect to ER and HER2 status by RNA-sequencing-based diagnostics. These patients might have benefited from an alternative treatment. Lacking a suitable ‘gold standard’ reference it is not possible to determine if sequencing-based classification provides more accurate results in this case. However, it has previously been reported that gene expression-based diagnostics is more prognostic than routine biomarkers^17^. Sequencing-based molecular profiling also provides a richer source of molecular data, and therefore has the potential resolve some ambiguous cases emerging in the current routine pathology setting, for example by allowing HER2 status to be determined by both RNA-sequencing and CNV profiling, and by utilizing multivariate biomarker panels for determining molecular subtypes (e.g. 50 genes in the PAM50 panel) rather then depending single markers (e.g. ER, PR, HER2) as is the case in traditional routine diagnostics. In this study 17 patients (5.5%) had somatic *ERBB2*(HER2) activating mutations, BRCA1 or BRCA2 mutations detected by panel DNA sequencing, which could impact on treatment. Further 162 patients (53%) had mutations or copy number alterations detected by DNA sequencing, which were specified in ongoing or concluded breast cancer clinical trials, indicating potential future benefit of sequencing-based diagnostics in a large proportion of patients. In addition to actionable somatic alterations, molecular subtyping and improvement in tumor grading systems has the potential to provide benefits for even larger groups of patients in the future.

The present study has some limitations; Firstly, the study is based on retrospective biobanked material and results have only been replicated in a single external study, consequently the results should be validated in a prospective setting prior to clinical implementation. Secondly, the study is not fully representative for the smallest sized tumors, as few small tumors were available for biobanking. However, we do not expect this to impact the interpretation of our results since the focus was on assessing concordance between routine diagnostic biomarkers and sequencing-based diagnostics. In this study a 10% cutoff for positive ER status was applied, in contrast to the 1% cutoff recommended in current ASCO-CAP guidelines, as there is currently lack of prospective randomized studies demonstrating benefits of a 1% cutoff. However, it has been reported that very few ER positive patients have <10% of cells staining positive^2^, suggesting that the actual impact of a different cutoff <10% is expected to be minor. Among the main benefits of sequencing-based diagnostics is the ability to detect germline alterations affecting drug metabolism, for example in the Dihydropyrimidine Dehydrogenase gene (*DPYD*)^43^,^44^. In future clinical implementations it would be highly relevant to ensure inclusion of additional relevant *DPYD* variants as well as other clinically relevant pharmacogenomics loci. The present study was based on fresh frozen (FF) tumor tissue, while formalin-fixed paraffin embedded (FFPE) material is standard in the routine clinical setting. Although there are quality differences FFPE and FF material, multiple publications demonstrate preserved gene expression profiles between FFPE and FF tissues, either by sequencing or other orthogonal technologies^45^,^46^. In practice, another alternative is to use preservatives, such as RNAlater to enable high quality RNA profiles^47^.

Our results revealed that breast cancer classification by sequencing-based molecular profiling is highly concordant with current routine diagnostic biomarkers and therefore has the potential to replace current generation routine biomarker assays in the clinic in the near future for a majority of patients. Sequencing-based molecular characterization also provides additional molecular information with implication on the choice of therapy such as molecular subtype and detection of somatic mutations in key genes including *BRCA1* and *ERBB2* (HER2). Furthermore RNA sequencing enabled us to dichotomize patients into high and low tumor grade, which has also previously been proposed^28^,^42^, but is not yet in clinical routine management. It is our intention to validate the results from this study through analysis of further 500 patients in a prospective study.

## Additional Information

**How to cite this article**: Rantalainen, M. *et al*. Sequencing-based breast cancer diagnostics as an alternative to routine biomarkers. *Sci. Rep.* **6**, 38037; doi: 10.1038/srep38037 (2016).

**Publisher's note:** Springer Nature remains neutral with regard to jurisdictional claims in published maps and institutional affiliations.

## Supplementary Material

Supplementary Material

## Figures and Tables

**Table 1:** Histopathological re-examination results for individuals discordant for ER and HER2 status between routine pathology and sequencing-based analysis.

Medical record				Seq-based characterization				Re-examination by IHC/SISH					
Patient ID	Discordant marker	ER	HER2	ER	HER2	HER2 copy-number*	subtype	ER%	HER2 IHC	ER	HER2	HER2 SISH	Concordant after re-examination
RDK1	ER	+	−	−	−		Basal	0	0	−			Y
RDK2	ER	−	−	+	−		LumA	100	0–1+	+			Y
RDL1	ER	+	−	−	−		Basal	0	0	−			Y
RDL2	ER	+	−	−	−		Basal	0	0	−			Y
RDL3	ER	+	−	−	−		Her2	0	2+	−			Y
RDL4	ER	+	+	−	+		Her2	0	3+	−			Y
RDK3	ER	+	+	−	+		Her2	20	3+	+			N
RDL5-a	ER	+	NA	−	+		Her2	20	3+	+			N
RDL5-b	ER	+	NA	−	+		Her2	80	2+	+			N
RDL6	ER	+	−	−	−		Her2	15	0	+			N
RDL7	ER/HER2	+	−	−	+	low amp	LumA	100	1+	+	−	NA	N/N
RDL8-a	HER2	+	+	+	−	low amp	LumB	80	0	+	−	NA	Y
RDL9	HER2	−	−	−	+	high amp	Her2	0	3+	−	+	NA	Y
RDL10	HER2	+	+	+	−	low amp	LumB	100	1+	+	+	positive	N
RDL8-b	HER2	+	+	+	−	low amp	LumB	90	1+	+	+	positive	N
RDL11	HER2	+	+	+	−	low amp	LumB	85	1+–2+	+	+	positive	N
RDL12	HER2	+	+	+	−	low amp	LumB	100	3+	+	+	NA	N

^*^See Supplemental Figures 3–8.

NA = missing/unavailable data.

Patients RDL5 and RDL8 had two separate tumor pieces re-examined by IHC/SISH (labeled as −a and −b)

(Key: LumA = Luminal A, LumB = Luminal B, Her2 = Her2-enriched, Basal = Basal-like).
