# Supplementary material for: Sequencing-based breast cancer diagnostics as an alternative to routine biomarkers
Source: Sci Rep. 2016 Nov 30;6:38037. doi: 10.1038/srep38037 (PMC5128815; doi:10.1038/srep38037)
Supplement: Supplementary Material [file srep38037-s1.pdf]

Supplemental Material

Sequencing-based breast cancer diagnostics as an alternative to routine biomarkers

Authors:

Mattias Rantalainen[1,a], *Daniel Klevebring*[1,a], *Johan Lindberg*[1,a], *Emma Ivansson*[1], Gustaf Rosin[2], *Lorand Kis*[2,3], *Fuat Celebioglu*[4], *Irma Fredriksson*[5,6], *Kamila Czene*[1], *Jan Frisell*[5,6], *Johan Hartman*[2,3], *Jonas Bergh*[2,3,b], *Henrik Grönberg*[1,b,*]

Affiliations:
[1] Department of Medical Epidemiology and Biostatistics, Karolinska Institutet, Stockholm, Sweden
[2] Department of Oncology-Pathology, Karolinska Institutet, Stockholm, Sweden
[3] Department of Clinical Pathology and Cytology, Radiumhemmet, Karolinska University Hospital, Stockholm, Sweden
[4] Department of Clinical Science and Education, Karolinska Institutet, Södersjukhuset, Stockholm, Sweden
[5] Department of Molecular Medicine and Surgery, Karolinska Institutet, Stockholm, Sweden
[6] Department of Breast- and Endocrine Surgery, Karolinska University Hospital, Stockholm, Sweden
[a] These authors contributed equally
[b] These authors contributed equally

* Corresponding author: Department of Medical Epidemiology and Biostatistics, Karolinska Institutet, Box 281,171 77 Stockholm, Sweden; Email: Henrik.Gronberg@ki.se; Phone: +46852482347; Fax: +468314975

List of contents

- Supplemental Methods (page 3 – page 10)
- Supplemental Table 1 (page 11). Summary of clinical phenotypes in the ClinSeq study.
- Supplemental Table 2 (page 12). List of gene ids of transcripts included in the transcriptomic grade model.
- Supplemental Figure 1 (page 17). Consort diagram of ClinSeq study.
- Supplemental Figure 2 (page 19). Technical reproducibility of RNAseq expression.
- Supplemental Figure 3 (page 20). Copy number profile of patient RDL10.
- Supplemental Figure 4 (page 20). Copy number profile of patient RDL8.
- Supplemental Figure 5 (page 21). Copy number profile of patient RDL9.
- Supplemental Figure 6 (page 21). Copy number profile of patient RDL7.
- Supplemental Figure 7 (page 22). Copy number profile of patient RDL12.
- Supplemental Figure 8 (page 22). Copy number profile of patient RDL11.
- Supplemental Figure 9 (page 23). Potential actionable mutations in the ClinSeq study.

Supplemental Methods

Study populations

Libro1

Biobanked tissue from patients participating in the Libro1 study[1] was used. The study was approved by the Regional Ethical Review Board in Stockholm (Sweden) with registration numbers 2009/254-31/4 and amendment 2012/465-32. All participants signed informed consent allowing for molecular profiling. Briefly, in 2009 women diagnosed with breast cancer between 2001 and 2008 in the Stockholm/Gotland regions and still alive in 2009 were asked to fill out a questionnaire and donate a blood samples to the study. Approximately 5500 women opted to participate. Of those participants who underwent primary surgery at the Karolinska University Hospital, we investigated if snap-frozen tumour was available in the Karolinska University Hospital breast cancer biobank. We received permission to withdraw tissue from the biobank for 279 out of these patients.

KARMA Tissue

During 2012, women who underwent primary surgery for breast cancer were prospectively asked to participate in the KARMA Tissue study (Stockholm, Sweden). The study was approved by the Regional Ethical Review Board in Stockholm (Sweden) with registration number 2010/958-31/3 and amendment 2011/765-3. All participants signed informed consent allowing for molecular profiling. Participants donated a blood sample, filled out a questionnaire and when possible, tumour tissue from the surgical specimen was snap-frozen on dry ice. In total, 108 patients we're enrolled in the

KARMA Tissue study, 82 out of these had been enrolled and had samples already collected at the Biobank at the time of sample retrieval for this study.

TCGA breast cancer

TCGA breast cancer data set: Clinical data from the TCGA invasive breast carcinoma dataset (provisional) was downloaded from the TCGA data portal (https://tcga-data.nci.nih.gov/tcga/) on 11th of December 2013 and included data for 1148 individuals. Unaligned RNAseq data from the TCGA dataset was subsequently downloaded (June 2014) after approval from the TCGA data access committee (N = 1126, all available individuals with unaligned data). A total of 1073 individuals were available with both unaligned RNAseq data and clinical data. RNAseq data was preprocessed as described in the section “RNASeq low-level processing”. Out of 1073 observations, 35 observations were excluded as potential outliers based on inspection of Principal Component Analysis scores and residuals. A total of 885 of the 1038 individuals had molecular subtype (PAM50) assignments available. All remaining individuals classified as Normal-like subtype (N=105) were excluded as the clinical relevance for this subtype has been questioned[2] , 780 samples were available for subtype analysis. 507 out of 1038 individuals had histological grade (Nottingham Histologic Grade) available and were used for validation of the transcriptomic grade model. ER status was available for 739 individuals, PR status for 738 individuals and HER2 status for 731 individuals, which were included in the validation of receptor status prediction.

Tissue handling

Frozen tissue was embedded in OCT and sectioned. To determine that tumour cells were present in the material used for extraction, we took a 5 µm section for hematoxylin/eosin (HE) staining, followed by 400 µm for extraction and finally another 5 µm section for HE staining. This enabled us to assess the fraction of tumour cells on both sides of the 400 µm we used for extraction of DNA and RNA. Samples were included in the study if at least one section was estimated to have >=30% tumor content by means of visual inspection, mean tumor content was estimated to ~70% for individuals included in the study.

Sample preparation and sequencing

RNA and DNA were extracted from fresh frozen tumors using AllPrep DNA/RNA/Protein mini kit (Qiagen). RNA was assessed using bioanalyzer to ensure high quality (RIN > 8). One µg of total RNA was used for rRNA depletion using RiboZero (Illumina) and stranded RNAseq libraries were constructed using TruSeq Stranded Total RNA Library Prep Kit (Illumina). Tumour DNA and normal DNA extracted from blood samples was quantified with Qubit (Invitrogen). To build libraries for low-pass and panel sequencing, DNA was fragmented using columns 4-9 in an Episonic Multi-Functional Bioprocessor 1100 with the following settings: Amp 10, pulse ON/OFF: 30/15 s, runtime: 30 min. Fragmented and used for library preparation using ThruPlex-FT (Rubicon Genomics) with 500 ng DNA as input following the manufacturers instructions after which an aliquot was taken for low-pass whole genome sequencing. Barcoded libraries were pooled in sets of 12 samples, and sequence capture was performed using the EZ SeqCap kit (Roche Nimblegen) with a custom capture target set, as previously described [3]. Sequencing was performed on Illumina HiSeq 2500 at Science for Life

Laboratories. WGS libraries were sequenced to on average 0.5x coverage, captured libraries to around 150x average coverage and RNAseq libraries to a median of 33 million read-pairs per library (paired-end 2 x 101 bases). In total the in-house ClinSeq data set contained 318 individuals with RNAseq data (see Figure S1 for consort diagram). The mean and standard deviation of tumour size and Ki-67 score, as well as the distribution of ER, PR, HER2 and histological grade is provided in Table S1.

Clinical data on routine biomarkers

Information on ER, PR, HER2 and Ki-67 as well as histological grade was collected from medical records. ER and PR status were for most individuals assessed by immunohistochemistry (IHC), classifying tumors that showed staining in 10% or more cells as positive. For a subset of the older individuals the radioimmunoassay was used to assess ER and PR status, classifying tumors that had >0.05 fmol/ug DNA as positive. Regarding HER2, a tumour was classified as positive (amplified) if FISH results indicated amplification or, in the absence of FISH results, if the sample was graded 3+ by IHC. Ki-67 was assessed by IHC and medical records report Ki-67 either as “high”/”low” or as a percent value. For the tumors with reported percentage 20% was considered as the threshold for high proliferation. Grade (Nottingham Histologic Grade) was recorded as 1,2 or 3.

Histopathological re-examination

Re-examination of ER and HER2 was performed for individuals where the receptor status was found to be discordant (N=17 ER discordant, N=8 HER2 discordant) between sequencing-based assessment and routine pathology, and where biobanked

material was accessible for re-examination (N=11 (out of 17) ER and N=8 (out of 8) HER2). FFPE tissue were sectioned and stained according to the current protocol at the Laboratory of Clinical Pathology / Cytology at Karolinska University Hospital. HER2 status was classified as positive in the re-examination if IHC was scored as 3+, or if SISH was completed and positive.

Bioinformatic processing

Standard Illumina adapters (AGATCGGAAGAGCACACGTCTGAACTCCAGTCAC and AGATCGGAAGAGCGTCGTGTAGGGAAAGAGTGTA) were trimmed using skewer version 0.1.117 [4] with default parameters, for both single end and paired end data.

RNASeq low-level processing

Alignment was carried out using STAR aligner version 2.4.0e [5] with the following parameters: "--outSAMmapqUnique 50", to set the mapping quality of uniquely mapped reads to 50; "--outSAMunmapped Within", to include unmapped reads in the resulting SAM file; "--chimSegmentMin 20" to require that a minimum of 20 bases maps to each end of a chimeric transcript (output in a separate file) and "--outSAMattributes NH HI AS nM NM MD XS" to include additional attributes in the SAM file. PCR duplicates were marked but not removed, using Picard MarkDuplicates version 1.128 (http://broadinstitute.github.io/picard). Gene expression estimates were calculated with HTSeq count version 0.6.1 [6] with the following parameters: "--stranded=no" for TCGA, since the TCGA Breast Cancer RNAseq data is non-stranded or "--stranded=reverse" for KI data, and "--mode=intersection-nonempty" for counting reads. The RNAseq count data were normalised using the TMM method [7] provided in the *edgeR* package [8]. Gene expression values are expressed as $\log_2$(counts per million), abbreviated as log2(CPM).

Principal component analysis (PCA) was used to detect outliers in the RNAseq data. 11 observations were excluded as potential outliers based on inspection of Principal Component scores and residuals, leaving 307 samples for further analyses (see Figure S1 for consort diagram).

Panel sequencing and WGS low level processing

Alignment to GRCh37 in karyotypic order including decoy sequences and unplaced contigs (available at ftp://ftp.1000genomes.ebi.ac.uk/vol1/ftp/technical/reference/phase2_reference_assembly_sequence/) was carried out using bwa mem version 0.7.7 [9] separately for the tumour and normal, while setting the read groups appropriately, after which reads were sorted and converted to bam format. Resulting bam files for the tumour and normal were then merged, realigned across indels and base qualities were recalibrated using GATK version 3.3. Calling of somatic SNVs was carried out with MuTect version 1.1.6 [10] and small indels with pindel version 0.2.5a3 [11], converted to VCF using the pindel2vcf tool in the pindel package (separately versioned, we used 0.5.8) and filtered using the custom SomaticPindelFilter walker version 0.1.2 (commit 19647d9 available at https://github.com/dakl/gatk). Variants were annotated with snpEff version 4.0 [12]. Silent mutations, and those in Introns, RNA, UTRs, Flanks, IGRs, and the ubiquitous Targeted_Region were excluded. Germline variant calling, including both SNPs and small indels, was done with freebayes version v9.9.2 [13] in target regions on a single-sample basis. Multiallelic variants were split using vcf_parser version 1.4 (https://github.com/moonso/vcf_parser) with the --split flag and normalized using

bcftools norm version 1.2 (https://github.com/samtools/bcftools). Somatic copy number profiling was carried out using BICseq [14] in R version 3.1.1.

Statistical analyses and prediction modelling

Individual logistic regression models were fitted with ER, PR, HER2 status as response variable and the expression of each corresponding gene (*ESR1*, *PGR* and *ERBB2* (HER2) respectively) as the predictor. Ki-67 was modelled by a linear penalized regression model, *elastic net* [15,16], which combines L1 and L2 penalisation, using the transcriptome-wide expression data as predictors and the clinical %Ki-67 as the response variable. Molecular subtypes were assigned using the Nearest Shrunken Centroid (NSC) classifier based [17] on the PAM50 gene set described by Parker *et al.* [18]. NSC model parameters were estimated using the TCGA dataset (see previous section). To reduce any potential batch differences between the ClinSeq and TCGA datasets, the two datasets were pre-processed using the same bioinformatic pipeline and variables were mean-centered and scaled to unit variance. Prediction modelling of histological grade was carried out using the *elastic net* model[16] to classify tumors into `high' and `low' transcriptomic grade (TG), corresponding to Nottingham Histologic Grade 1 and 3. Microarray-based gene expression profiling has previously been applied to dichotomize tumour by their grade [19,20], here we apply a different statistical modelling framework and base our models on RNA sequencing data with similar objectives. Individual linear models (elastic net) were fitted for each subcomponent of the histological grade: mitotic count, nuclear atypia and tubular formation. Each of these models was trained on individuals with a clinical score of 1 or 3 for each respective component. For prediction of transcriptomic grade, the predicted score ($\hat{Y}$) for each component were combined into

an overall score defined by the sum over the predictions from each subcomponent model, $\hat{Y}$_mitotic (mitotic count), $\hat{Y}$_nuclearity (nuclear atypia), $\hat{Y}$_tubularity (tublar formation). Prediction performance of models were evaluated using a class-balance Monte-Carlo cross-validation procedure using 80% of the data as the training set in each cross-validation round for 50 rounds of cross-validation. To estimate prediction performance in the case of penalized regression models, a nested cross-validation procedure was implemented allowing for unbiased estimation of prediction performance while also optimizing model parameters empirically. Optimization of the amount of penalization (*lambda*) in each elastic net model was optimised in the inner cross-validation, using only the training data from the outer cross-validation. The parameter *alpha*, describing the relative weight between L1 and L2 penalisation was set to 0.5. The prediction performance was estimated using the test set in the outer cross-validation round, i.e. using data that were not involved in any part of the model optimization or parameter estimation. Validation of classification performance of ER, PR, HER2 status and transcriptomic grade in the TCGA data set were carried out by predicting TCGA samples using models fitted on the ClinSeq data set (variables in both data sets were mean centered and scaled to unit variance), based on the predictions, ROC curves and AUC were calculated for the TCGA data set. Estimation of ROC curves and AUC were carried out using the pROC [21] package for R, optimal decision boundaries for binary classification problems were determined by the point with minimal distance to the top-left corner of the ROC curve. All statistical analyses were carried out in the R environment [22].

Supplemental Tables

Variable	Mean	Sd	% Positive Status
Tumour size (mm)	26,21	17,01	
Histological grade (1/2/3)			14/44/42
IHC_ER			85
IHC_PR			64
IHC/FISH_HER2			16
IHC_KI67 (%)	28,87	24,06	

Supplemental Table 1. Summary of clinical phenotypes in the ClinSeq study.

gene id

ENSG00000170312
ENSG00000122952
ENSG00000175063
ENSG00000113368
ENSG00000250210
ENSG00000173715
ENSG00000119969
ENSG00000083312
ENSG00000173281
ENSG00000181061
ENSG00000169181
ENSG00000129219
ENSG00000123388
ENSG00000161800
ENSG00000104549
ENSG00000144182
ENSG00000117724
ENSG00000138160
ENSG00000104413
ENSG00000160584
ENSG00000136936
ENSG00000150938
ENSG00000179029
ENSG00000108590
ENSG00000081386
ENSG00000118193
ENSG00000099960
ENSG00000170222
ENSG00000013810
ENSG00000008311
ENSG00000112984
ENSG00000006625
ENSG00000109775
ENSG00000141295
ENSG00000135094
ENSG00000257335
ENSG00000109738
ENSG00000135842
ENSG00000144369
ENSG00000170615
ENSG00000157456
ENSG00000111602

ENSG00000090889
ENSG00000172748
ENSG00000126787
ENSG00000078579
ENSG00000136932
ENSG00000148773
ENSG00000136457
ENSG00000135476
ENSG00000197208
ENSG00000181273
ENSG00000174957
ENSG00000122592
ENSG00000008226
ENSG00000186115
ENSG00000152291
ENSG00000161904
ENSG00000215472
ENSG00000127564
ENSG00000166160
ENSG00000069011
ENSG00000224186
ENSG00000124092
ENSG00000203926
ENSG00000196406
ENSG00000177535
ENSG00000101407
ENSG00000187123
ENSG00000179097
ENSG00000183607
ENSG00000203818
ENSG00000167360
ENSG00000127995
ENSG00000166573
ENSG00000132155
ENSG00000151849
ENSG00000182645
ENSG00000140471
ENSG00000173273
ENSG00000239306
ENSG00000120915
ENSG00000175143
ENSG00000102225
ENSG00000101447

ENSG00000183475
ENSG00000170291
ENSG00000100104
ENSG00000120094
ENSG00000113209
ENSG00000119953
ENSG00000255181
ENSG00000188393
ENSG00000147434
ENSG00000182035
ENSG00000105695
ENSG00000203724
ENSG00000267368
ENSG00000105392
ENSG00000149269
ENSG00000165566
ENSG00000143443
ENSG00000070388
ENSG00000197013
ENSG00000173991
ENSG00000196230
ENSG00000112559
ENSG00000142619
ENSG00000167528
ENSG00000141279
ENSG00000099875
ENSG00000180611
ENSG00000186453
ENSG00000135097
ENSG00000079459
ENSG00000159915
ENSG00000206559
ENSG00000152763
ENSG00000111012
ENSG00000166796
ENSG00000131368
ENSG00000163507
ENSG00000188338
ENSG00000109685
ENSG00000256861
ENSG00000134207
ENSG00000033030
ENSG00000175229

ENSG00000258484
ENSG00000109436
ENSG00000221858
ENSG00000087460
ENSG00000154328
ENSG00000164756
ENSG00000255633
ENSG00000249931
ENSG00000244476
ENSG00000074211
ENSG00000167720
ENSG00000149782
ENSG00000178409
ENSG00000114654
ENSG00000146955
ENSG00000188379
ENSG00000129990
ENSG00000187151
ENSG00000075218
ENSG00000161509
ENSG00000153495
ENSG00000131080
ENSG00000273259
ENSG00000088305
ENSG00000130818
ENSG00000185262
ENSG00000167578
ENSG00000214860
ENSG00000165917
ENSG00000127954
ENSG00000184178
ENSG00000265264
ENSG00000164329
ENSG00000029993
ENSG00000178796
ENSG00000173894
ENSG00000174358
ENSG00000178922
ENSG00000180011
ENSG00000186185
ENSG00000268797
ENSG00000214456
ENSG00000204450

ENSG00000133937

ENSG00000172410

ENSG00000145632

ENSG00000186575

ENSG00000095627

ENSG00000171396

ENSG00000131899

ENSG00000254726

ENSG00000080546

ENSG00000164124

ENSG00000104228

ENSG00000080511

ENSG00000172058

ENSG00000130711

ENSG00000183246

ENSG00000155890

ENSG00000185798

ENSG00000130958

ENSG00000171595

ENSG00000268964

ENSG00000163882

ENSG00000147155

ENSG00000188004

ENSG00000153230

ENSG00000154485

ENSG00000254806

ENSG00000120948

ENSG00000102021

ENSG00000131378

ENSG00000183963

ENSG00000164334

ENSG00000126457

ENSG00000140505

ENSG00000171564

ENSG00000099840

ENSG00000255526

ENSG00000213934

ENSG00000206527

ENSG00000186871

ENSG00000177885

ENSG00000169704

ENSG00000165458

ENSG00000244623

ENSG00000101883
ENSG00000138346
ENSG00000163093
ENSG00000140479

Supplemental Table 2. List of gene ids (ensemble) of transcripts included in the transcriptomic grade model.

Supplemental Figures

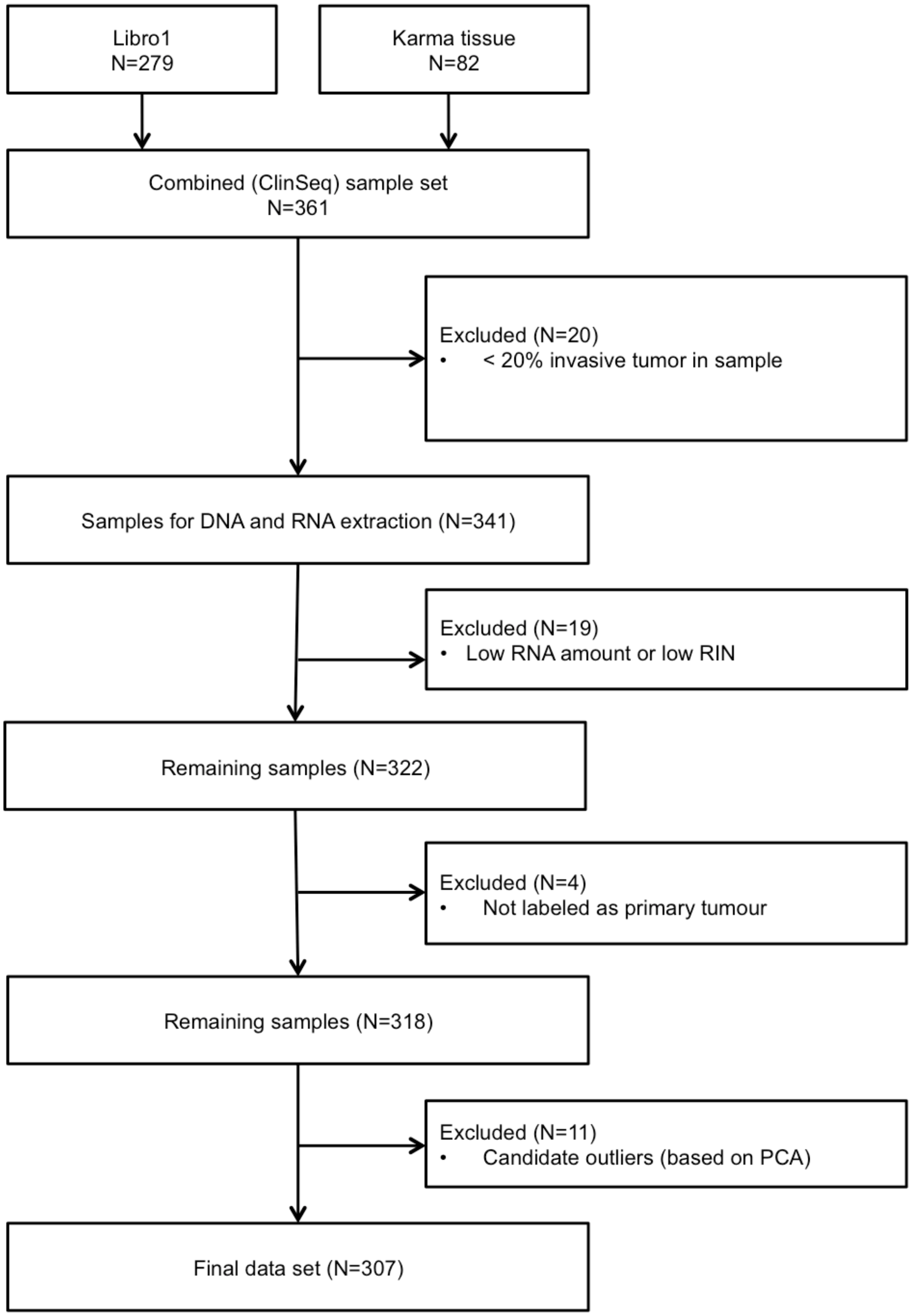

Supplemental Figure 1. Consort diagram of ClinSeq study.

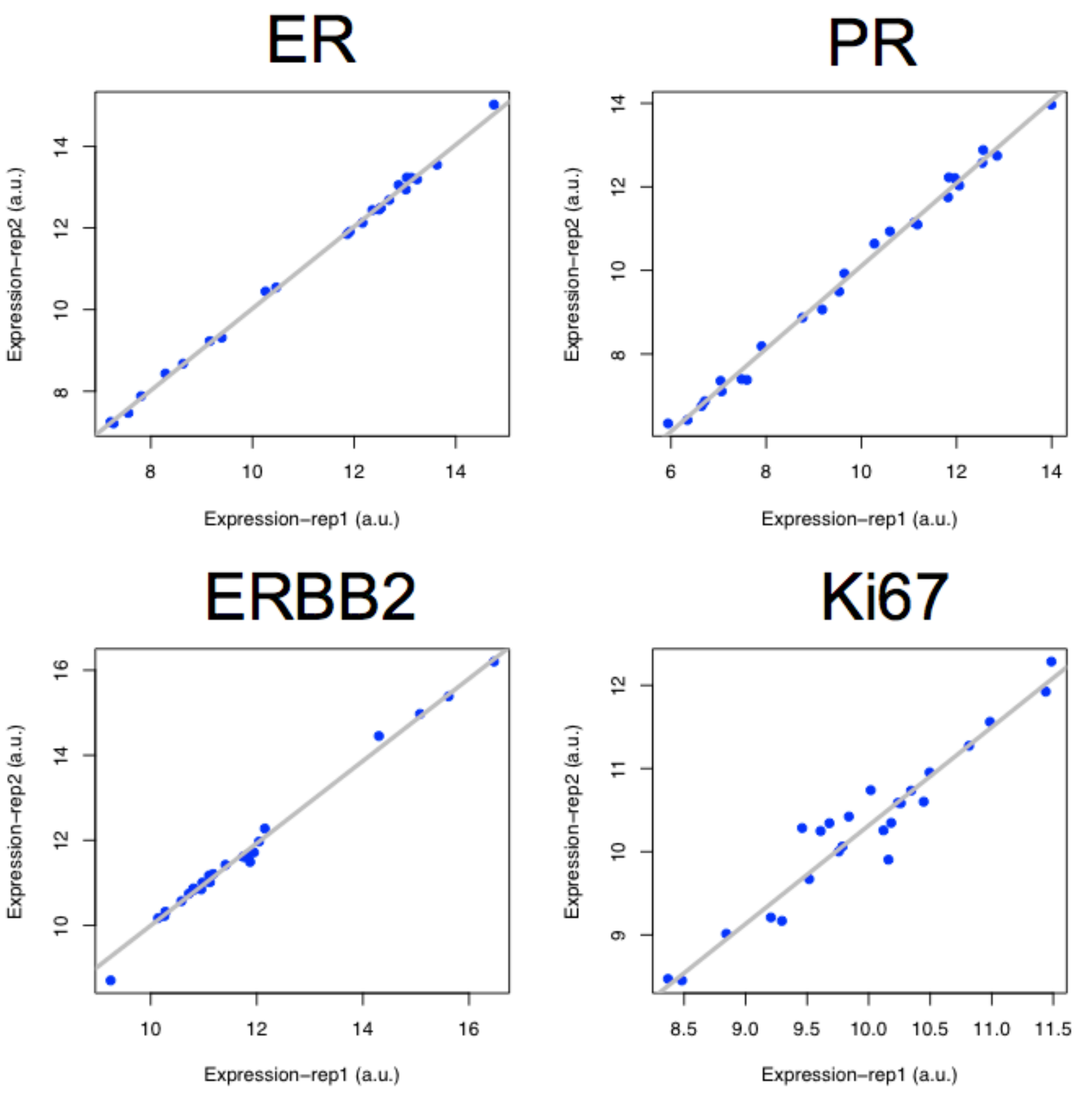

Supplemental Figure 2. Technical reproducibility of RNAseq expression levels for ER (ESR1), PR (PGR), *ERBB2*, and Ki67 based on N=20 technical duplicates.

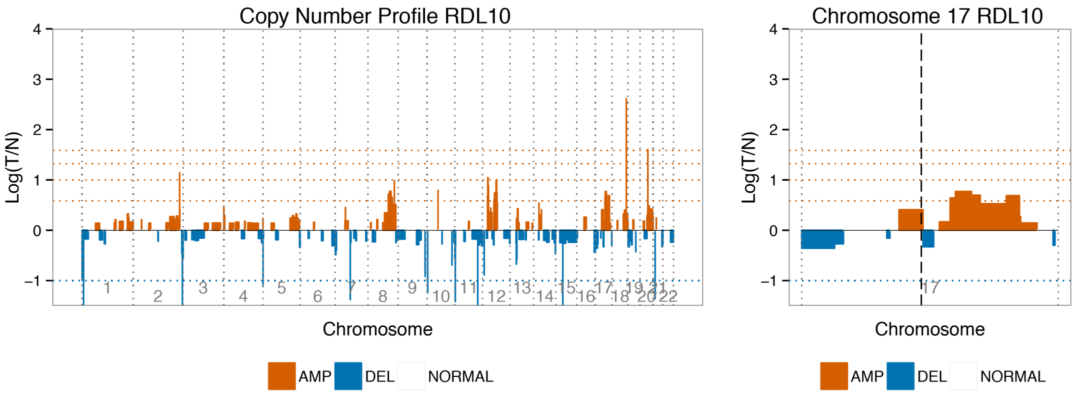

Supplemental Figure 3. Copy number profile across the genome (left) and at the location of *ERBB2* (dashed line).

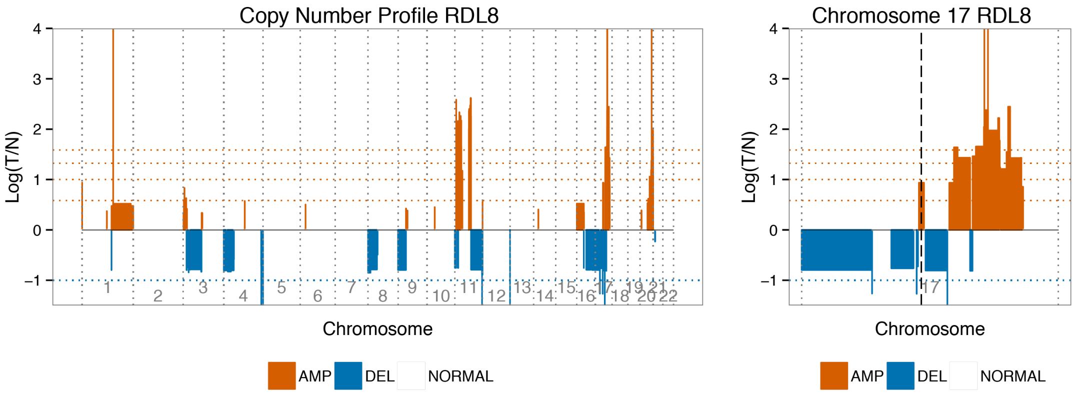

Supplemental Figure 4. Copy number profile across the genome (left) and at the location of *ERBB2* (dashed line).

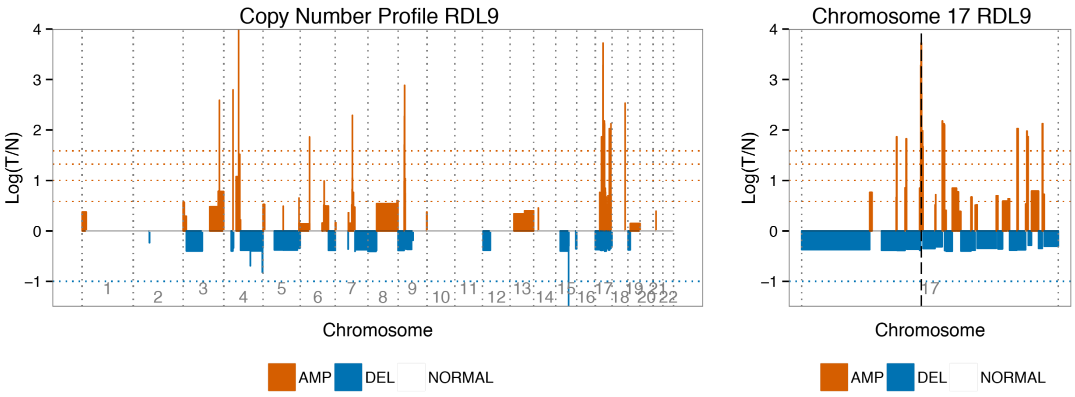

Supplemental Figure 5. Copy number profile across the genome (left) and at the location of *ERBB2* (dashed line).

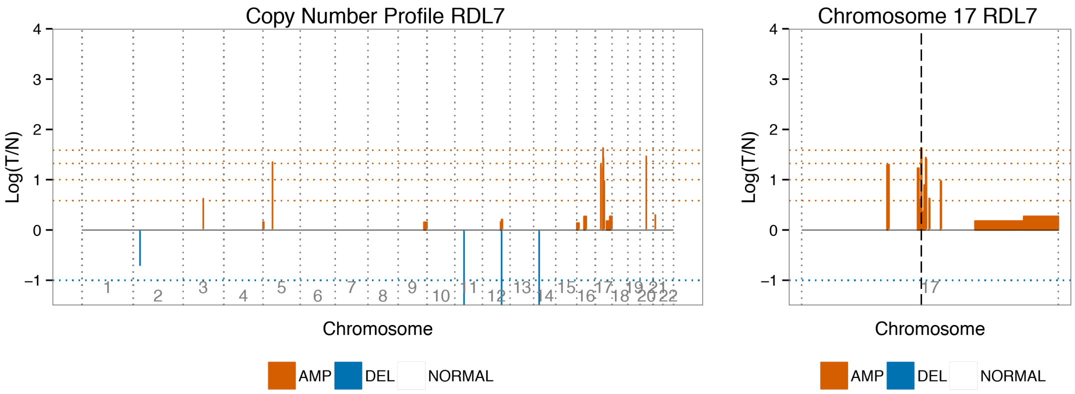

Supplemental Figure 6. Copy number profile across the genome (left) and at the location of *ERBB2* (dashed line).

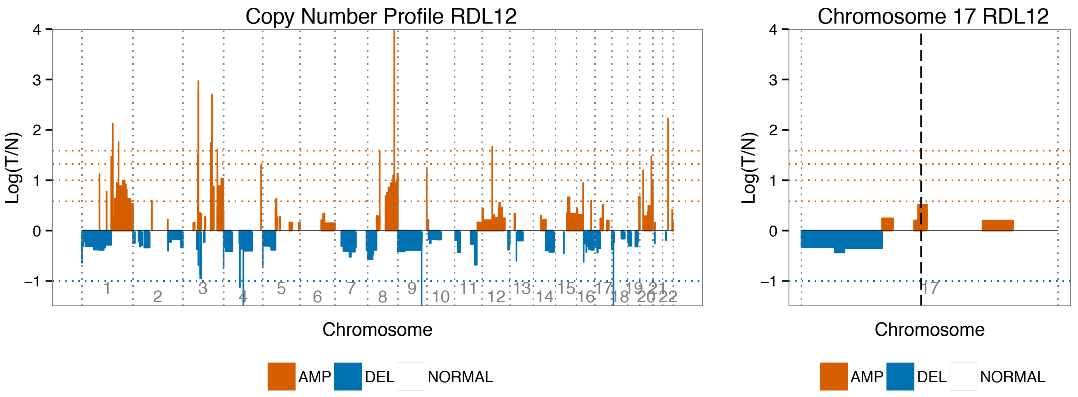

Supplemental Figure 7. Copy number profile across the genome (left) and at the location of *ERBB2* (dashed line).

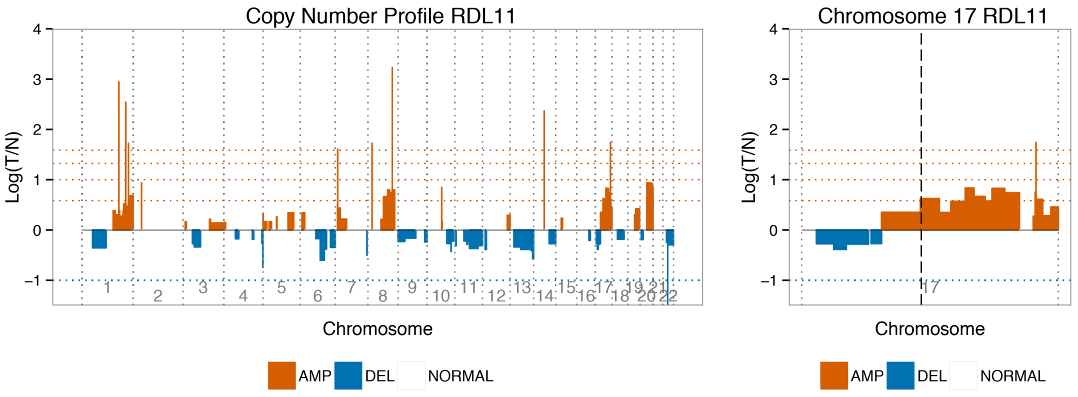

Supplemental Figure 8. Copy number profile across the genome (left) and at the location of *ERBB2* (dashed line).

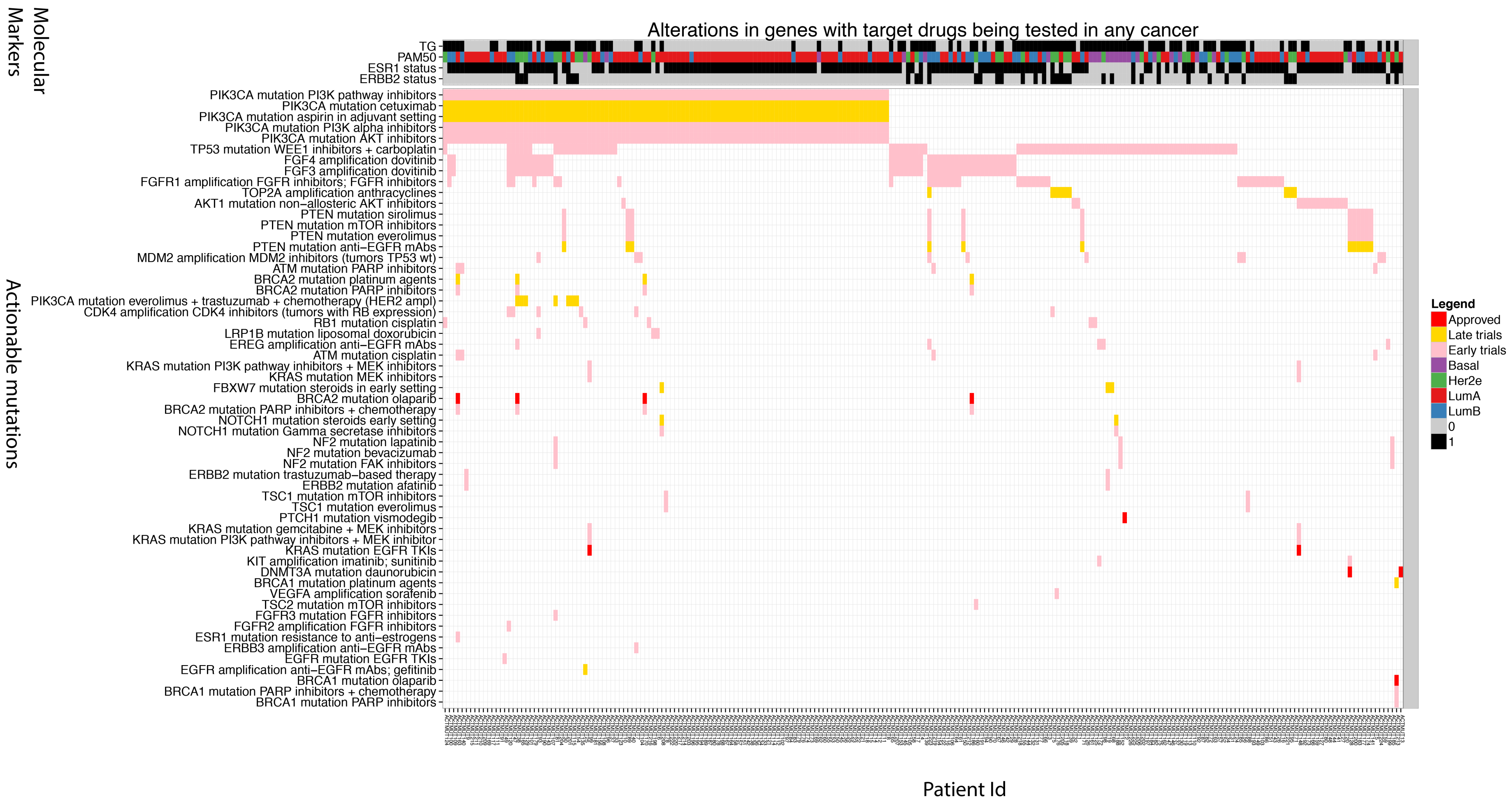

Alterations in genes with target drugs being tested in any cancer
Molecular Markers
TG
PAM50
ESR1 status
ERBB2 status
Actionable mutations
PIK3CA mutation PI3K pathway inhibitors
PIK3CA mutation cetuximab
PIK3CA mutation aspirin in adjuvant setting
PIK3CA mutation PI3K alpha inhibitors
PIK3CA mutation AKT inhibitors
TP53 mutation WEE1 inhibitors + carboplatin
FGF4 amplification dovitinib
FGF3 amplification dovitinib
FGFR1 amplification FGFR inhibitors; FGFR inhibitors
TOP2A amplification anthracyclines
AKT1 mutation non−allosteric AKT inhibitors
PTEN mutation sirolimus
PTEN mutation mTOR inhibitors
PTEN mutation everolimus
PTEN mutation anti−EGFR mAbs
MDM2 amplification MDM2 inhibitors (tumors TP53 wt)
ATM mutation PARP inhibitors
BRCA2 mutation platinum agents
BRCA2 mutation PARP inhibitors
PIK3CA mutation everolimus + trastuzumab + chemotherapy (HER2 ampl)
CDK4 amplification CDK4 inhibitors (tumors with RB expression)
RB1 mutation cisplatin
LRP1B mutation liposomal doxorubicin
EREG amplification anti−EGFR mAbs
ATM mutation cisplatin
KRAS mutation PI3K pathway inhibitors + MEK inhibitors
KRAS mutation MEK inhibitors
FBXW7 mutation steroids in early setting
BRCA2 mutation olaparib
BRCA2 mutation PARP inhibitors + chemotherapy
NOTCH1 mutation steroids early setting
NOTCH1 mutation Gamma secretase inhibitors
NF2 mutation lapatinib
NF2 mutation bevacizumab
NF2 mutation FAK inhibitors
ERBB2 mutation trastuzumab−based therapy
ERBB2 mutation afatinib
TSC1 mutation mTOR inhibitors
TSC1 mutation everolimus
PTCH1 mutation vismodegib
KRAS mutation gemcitabine + MEK inhibitors
KRAS mutation PI3K pathway inhibitors + MEK inhibitor
KRAS mutation EGFR TKIs
KIT amplification imatinib; sunitinib
DNMT3A mutation daunorubicin
BRCA1 mutation platinum agents
VEGFA amplification sorafenib
TSC2 mutation mTOR inhibitors
FGFR3 mutation FGFR inhibitors
FGFR2 amplification FGFR inhibitors
ESR1 mutation resistance to anti−estrogens
ERBB3 amplification anti−EGFR mAbs
EGFR mutation EGFR TKIs
EGFR amplification anti−EGFR mAbs; gefitinib
BRCA1 mutation olaparib
BRCA1 mutation PARP inhibitors + chemotherapy
BRCA1 mutation PARP inhibitors
Patient Id
Legend
Approved
Late trials
Early trials
Basal
Her2e
LumA
LumB
0
1

Supplemental Figure 9. Potential actionable mutations in the ClinSeq study. Each patient's mutational profile (y-axis, Patient Id) was matched to the Dienstmann *et al.* knowledge base of actionable mutations including all trials, excluding drugs targeting ERBB2 amplification. Trials (x-axis) are classified as "Early" or "Late" (color coded as pink or yellow), or 'Approved' (color coded as red) from the Dienstmann *et al.* knowledge base[23]. Presence of a colored marker indicates that the mutational profile of the Clinseq tumor (y-axis rows, Patient Id) match the actionable mutation in a trial (x-axis, Actionable mutations). The leftmost vertical tracks provide information about molecular markers, including transcriptomic grade (TG), molecular subtype (PAM50), estrogen receptor status (ESR1) and HER2 status (ERBB2) for each tumor in the study.

REFERENCES

1. Holm J, Humphreys K, Li J, et al: Risk factors and tumor characteristics of interval cancers by mammographic density. J Clin Oncol 33:1030-7, 2015
2. Eroles P, Bosch A, Perez-Fidalgo JA, et al: Molecular biology in breast cancer: intrinsic subtypes and signaling pathways. Cancer Treat Rev 38:698-707, 2012
3. Lindberg J, Klevebring D, Liu W, et al: Exome sequencing of prostate cancer supports the hypothesis of independent tumour origins. Eur Urol 63:347-53, 2013
4. Jiang H, Lei R, Ding SW, et al: Skewer: a fast and accurate adapter trimmer for next-generation sequencing paired-end reads. BMC Bioinformatics 15:182, 2014
5. Dobin A, Davis CA, Schlesinger F, et al: STAR: ultrafast universal RNA-seq aligner. Bioinformatics 29:15-21, 2013
6. Anders S, Pyl PT, Huber W: HTSeq-a Python framework to work with high-throughput sequencing data. Bioinformatics 31:166-9, 2015
7. Robinson MD, Oshlack A: A scaling normalization method for differential expression analysis of RNA-seq data. Genome Biol 11:R25, 2010
8. Robinson MD, McCarthy DJ, Smyth GK: edgeR: a Bioconductor package for differential expression analysis of digital gene expression data. Bioinformatics 26:139-40, 2010
9. Li H, Durbin R: Fast and accurate short read alignment with Burrows-Wheeler transform. Bioinformatics 25:1754-60, 2009
10. Cibulskis K, Lawrence MS, Carter SL, et al: Sensitive detection of somatic point mutations in impure and heterogeneous cancer samples. Nat Biotechnol 31:213-9, 2013
11. Ye K, Schulz MH, Long Q, et al: Pindel: a pattern growth approach to detect break points of large deletions and medium sized insertions from paired-end short reads. Bioinformatics 25:2865-71, 2009
12. Cingolani P, Platts A, Wang le L, et al: A program for annotating and predicting the effects of single nucleotide polymorphisms, SnpEff: SNPs in the genome of Drosophila melanogaster strain w1118; iso-2; iso-3. Fly (Austin) 6:80-92, 2012
13. Garrison E, Marth G: Haplotype-based variant detection from short-read sequencing. arXiv preprint arXiv:1207.3907, 2012
14. Xi R, Hadjipanayis AG, Luquette LJ, et al: Copy number variation detection in whole-genome sequencing data using the Bayesian information criterion. Proc Natl Acad Sci U S A 108:E1128-36, 2011
15. Zou H, Hastie T: Regularization and variable selection via the elastic net. Journal of the Royal Statistical Society Series B-Statistical Methodology 67:301-320, 2005
16. Friedman J, Hastie T, Tibshirani R: Regularization Paths for Generalized Linear Models via Coordinate Descent. J Stat Softw 33:1-22, 2010
17. Tibshirani R, Hastie T, Narasimhan B, et al: Diagnosis of multiple cancer types by shrunken centroids of gene expression. Proc Natl Acad Sci U S A 99:6567-72, 2002
18. Parker JS, Mullins M, Cheang MC, et al: Supervised risk predictor of breast cancer based on intrinsic subtypes. J Clin Oncol 27:1160-7, 2009
19. Ivshina AV, George J, Senko O, et al: Genetic reclassification of histologic grade delineates new clinical subtypes of breast cancer. Cancer Res 66:10292-301, 2006
20. Sotiriou C, Wirapati P, Loi S, et al: Gene expression profiling in breast cancer: understanding the molecular basis of histologic grade to improve prognosis. J Natl Cancer Inst 98:262-72, 2006
21. Robin X, Turck N, Hainard A, et al: pROC: an open-source package for R and S+ to analyze and compare ROC curves. BMC Bioinformatics 12:77, 2011

22. R Core Team: R: A language and environment for statistical computing. Vienna, Austria, 2013

23. Dienstmann R, Jang IS, Bot B, et al: Database of genomic biomarkers for cancer drugs and clinical targetability in solid tumors. Cancer Discov 5:118-23, 2015
